# Predictors of Post-stroke Cognition Among Geriatric Patients: The Role of Demographics, Pre-stroke Cognition, and Trajectories of Depression

**DOI:** 10.3389/fpsyg.2021.717817

**Published:** 2021-07-26

**Authors:** Christiana Kang

**Affiliations:** Basis International School Guangzhou, Guangzhou, China

**Keywords:** stroke, depression, cognitive impairment, geriatric population, post stroke depression

## Abstract

Stroke is a prevalent disease among geriatric population, which tends to deteriorate cognitive ability and mental health. In such context, cognitive impairment and geriatric depression generate mutually deteriorating impacts on each other. Using the Health and Retirement Study, this study examined depression and cognition before, immediately after, and 2 years after the onset of stroke. Through latent growth mixture modeling, four different trajectories of depression were identified: resilience, recovery, emergent depression, and chronicity. We used demographics including gender, age, race, and ethnicity, activity of daily life, baseline cognition, and trajectories of depression to predict cognitive ability 2 years after the stroke. Both aforementioned demographic factors and pre-stroke cognition were predictive of post-stroke cognition, but the inclusion of depression trajectories further improved the predictive ability. Emergent depression and chronicity were two significant predictors of worse post-stroke cognition. This study showed the importance of considering a more specific trajectotrial interrelationship between depression and cognition in geriatric stroke patients.

## Introduction

Stroke is a significant life-threatening, neurological disease that accounts for approximately 1 of every 19 deaths in the United States (Yousufuddin and Young, 2019; Virani et al., 2020). In the United States, stroke is much more prevalent among geriatric population, with a 10% difference between the prevalence rate of the age group of 20–39 and that of those older than 80 (Virani et al., 2020). The probability of stroke doubles with each decade after the age of 45 and more than 70% of all strokes occurs after the age of 65 (Kelly-Hayes, 2010). The literature calls special attention to the impact of stroke among the geriatric population. Accompanied by the natural deterioration of cognitive function due to aging, elder people are more prone to depression and cognitive impairment following stroke. Depression and cognitive impairment affect about 30–50% stroke survivors, respectively (Towfighi et al., 2016). Therefore, it is critical and pressing to understand the patterns and underlying mechanisms of cognition in relation to geriatric depression in the context of stroke. Understanding the relationship between cognition and depression among stroke survivors not only provides insight into the cognitive and emotional well-being among this population but also informs corresponding cognitive remediation and psychotherapy programs that can best address these two interrelated challenges that stroke patients face.

### Impact of Stroke on Cognition and Depression

Stroke has a negative impact on cognitive performance in terms of one or multiple cognitive domains, such as attention, memory, language, and spatial and temporal orientation. With an aging population because of the increasing life expectancy and decreasing mortality rate due to improved medical treatment, the rate of post-stroke cognitive impairment is on the rise. It is, therefore, important to understand the influence strokes have on cognitive function since studies report that more than half of stroke survivors presents new onset or aggravated cognitive deficits after strokes (Donovan et al., 2008). Specifically, memory is an important domain of cognitive function for the evaluation of stroke's impact on cognition as the prevalence of memory problems in elder stroke survivors varies from 23 to 55% (Al-Qazzaz et al., 2014). According to these authors, brain infarcts can injure a particular neuroanatomical location (e.g., hippocampus, whose communication with neural network memory processing depends on) or parts of the neural network. Thus, brain infarcts are assumed to contribute to poorer memory and cognitive performance based on their close association (Blum et al., 2012). A study conducted by Comijs et al. and colleagues 2009 reveals the significant decline in memory in the form of immediate and delay recall as well as in information processing speed following a stroke.

In additions to memory, stroke can also bring detrimental effect on the mental status of geriatric patients, another vital measure of cognitive function, due to their specific or generalized effects on the brain. Stroke is one of the recognized causes of delirium, a type of mental confusion and emotional disruption (McManus et al., 2007). In a study focusing on stroke patients with a mean age of 62, 7% of the patients experienced altered mental status caused by stroke. Patients with post-stroke delirium had distraction, decreased consciousness, and disorganized thinking, which all indicates the mental status disturbance after a stroke leads to cognitive dysfunction (Wilber, 2006).

The association of stroke with depression suggests harmful impacts of stroke on mental health. One third of the stroke patients experience Post Stroke Depression (PSD) (Hommel et al., 2015; Santos et al., 2016). Elder PSD patients undergo a sense of decreasing life fulfillment via subjective complaints of their impaired cognitive function due to stroke, such as poorer memorization ability and deficiency of concentration, though elder people did not feel hopeless about their future, which is common among younger patients (Lokk and Delbari, 2010). The significant scope of PSD and its underlined psychological reason indicate the negative impact stroke brings on depression.

### Cognition and Depression in the Context of Stroke

Cognitive impairment and depression are connected and reciprocally influential in the context of stroke. Modeled with binomial regressions, it shows as post-stroke cognitive impairment worsens, depression gradually increases. Conversely, stroke patients who are depressed perform more poorly than those who are not depressed on neuropsychological tests of a variety of cognitive function domains, including general functions, memory, and executive functions (Hommel et al., 2015). Stroke patients with comorbid depression performed noticeably worse on global cognitive tests than stroke patients without depression (Tang et al., 2018). Particularly, those with higher scores on the Geriatric Depression Scale (GDS) exhibited noticeable post-stroke decline in memory function measured by the Montreal Cognitive Assessment (Tene et al., 2016). Besides, stroke patients who were depressed were more likely to experience intrusive memory than non-depressed stroke patients (Sampson et al., 2003).

Such mutually negative effect is also observed in the relation between mental status and depression. Depression deteriorates recovery in mental status and cognitive performance, as there is an approximately 30% difference in the improvement rate of depressed patients and non-depressed patients (Morris et al., 1992). Domains of mental status, such as speech disturbance and impaired consciousness, contribute to and further help predict the development of PSD (Towfighi et al., 2016). Poorer cognitive performance in other non-memory domains, such as executive function, visuospatial ability, and attention, was also observed in those who had higher GDS scores (Tene et al., 2016).

### Research Gap in the Literature

The investigation of cognition and depression in the context of stroke has been limited as many studies thus far failed to recruit representative samples, adopted cross-sectional designs, and had relatively small sample sizes. As studies showcase that stroke location does not correct with depression, the core interest has shifted from the question of which part of the brain interrupted by stroke increases the risk of depression or damages cognitive functions toward the exploration of more detailed relation between post-stroke depression and cognitive ability (Berg et al., 2003; Hommel et al., 2015). However, many studies focus on a specialized unit of stroke patients for investigation of the relationship between depression and cognition under the impact of stroke. Subsequently, the extrapolation of these studies' results to the population of general geriatric stroke patients is not preferable because characteristics of aging, severe strokes, and prestrike conditions are not specifically included while being limited by the location of study conducted (Hommel et al., 2015; Kapoor et al., 2019). Furthermore, the small sample size in most studies with about 200 participants further restricts generalization. In addition, the period of patients suffering from PSD a majority of studies target is limited to a period under 5 years due to the restraint of individual researchers' time and financial restraints (Berg et al., 2003).

### Goal of the Study

To address the gap that existed in the literature, this study used data from the Health and Retirement Study (HRS). HRS is a longitudinal, multidisciplinary household survey conducted by the Institute for Social Research at the University of Michigan that aims to investigate different aspects of aging population in US. The prospective nature of HRS gives it the advantage of exploration of the relation between cognition and late-in life depression over other datasets as it includes the pre-stroke conditions for more accurate contrast within individuals to evaluate the baseline's impact. With a relatively big sample size, a variety of geriatric patients can be investigated, which thus helps control potential confounding variables. This study analyzed and modeled the cognition and depression relation in the context of stroke experienced by aged population; explored the influence of different trajectories of the geriatric PSD on cognitive impairment.

## Method

### Data and Participants

The current study used data from the Health and Retirement Study (HRS). HRS data includes observation in individual respondent, household-level variables, and information on spouses. HRS data were extracted from the RAND HRS Fat Files of each survey wave (University of Michigan National Institute of Aging, 2020). We identified participants who experienced incidents of stroke, and for participants who experienced multiple strokes, we only included the first onset of stroke. Given our interest in cognition and depression before, during, and after stroke, we included three waves of data for each eligible participant: before the report of stroke (i.e., pre-stroke), when the participant reported stroke (i.e., stroke), and following the participant's report of stroke (i.e., post-stroke). For this study, we excluded participants that experience stroke at the first three waves, because cognition data in these waves were either different or lacked mental status measurement. The final sample included a total of 766 participants (337 men, 429 women) averaging 77.35 years old (SD = 6.54), who reported the wave that they experience stroke for the first time. The sample resembles the US population with 84.6% White or Caucasian, 14.5% Black or African American, and 0.9% other races.

### Measures

We included the following variables in our analyses: gender (RAGENDER), age at the stroke (RwAGEY_E), race (RARACEM), ethnicity (RAHISPAN), cognition (RwCOGTOT), activity of daily living (RwADLA), and depression (RwCESD). Gender was dummy coded such that 1 is male and 0 is female. Race was dummy coded such that 0 is Caucasian or White, and 1 represents Non-White. Ethnicity was dummy coded with 0 representing non-Hispanic and 1 representing Hispanic. Cognition was evaluated in the form of a total cognition score that sums the total recall and mental status indices, which was gained by the test on immediate and delayed word recall, the serial 7s test, counting backwards, naming tasks, and vocabulary (University of Michigan National Institute of Aging, 2020). Activity of daily living contributes to a multi-aspect understanding of the stroke patients' cognition performance, as physical disability can exemplify a form of cognitive impairment. Depression was measured by score on the Center for Epidemiologic Studies Depression (CESD) scale. The CESD score is the result of six “negative” indicators (depression, everything is an effort, sleep is restless, felt alone, felt sad, and could not get going) minus two “positive” indicators (felt happy and enjoyed life for all or most of the time) (University of Michigan National Institute of Aging, 2020).

### Data Analytic Plan

Analyses were performed in R version 4.0.3 (R Project for Statistical Computing). We employed the baseline floating approach to examine cognition and depression in the context of stroke. We first performed descriptive data analysis to understand whether stroke has an impact on the development of depression and cognition or not. Concerning the group differences in patterns of development of depression, we use the person-centered approach with the application of the latent growth mixture model to identify the predominant patterns of depression trajectories. This was performed using Mplus 7.4 (Muthén and Muthén, 2019) to identify the best fitting trajectory models for depressive symptoms. Growth mixture modeling does not rely on the assumption that individuals can meaningfully described by a homogeneous mean response. Instead, it allows to tease out subpopulations characterized by different growth patterns (i.e., heterogeneity). In our analysis, we compared models with intercept, slope, and quadratic parameters as either fixed or random. In our final model, we allowed both intercept and slope to be freely estimated. Given that the quadratic term was insignificant, we excluded it in the final model. The best class solution was determined by fit indices including the Akaike Information Criteria (AIC), Bayesian Information Criteria (BIC), sample-size adjusted Bayesian Information Criteria (SSABIC), Entropy, and Lo-Mendell-Rubin Adjusted Likelihood Ratio Test (LMR LRT). Finally, we used demographic, activity of daily life, baseline cognition, and trajectory of depression to predict cognition 2 years after the stroke.

## Results

### Descriptive Statistics

Table 1 shows the descriptive statistics of major variables of interest. The sample was generally old (mean age M = 77, SD = 2.01). The pre-depression score was 1.89 on average (SD = 2.01). Depression in the wave when the participants reported stroke was slightly elevated (For Time 2, M = 1.98, SD = 2.01), but over time, depression flattened out (For Time 3, M = 1.99, SD = 2.03). The pre-cognition score was on average 20.96 (SD = 5.16). Cognition declined immediately following the stroke (For Time 2, M = 1.98, SD = 2.01), though it slowed to decrease over time (For Time 3, M = 1.99, SD = 2.03). As the score for depression on average increased and the score for cognition averagely decreased, stroke had a harmful effect on depression and cognition.

**Table 1:** Descriptive statistics for continuous variables.

**Variable**	**M**	**SD**	**Min**	**Max**
Age	77.35	6.54	56.00	98.00
Pre-stroke Cog	20.96	5.16	4.00	32.00
Pre-stroke Dep	1.89	2.01	0.00	8.00
Pre-stroke ADL	0.56	1.05	0.00	5.00
Stroke-onset Cog	19.93	5.38	2.00	32.00
Stroke-onset Dep	1.98	2.01	0.00	8.00
Stroke onset ADL	0.75	1.19	0.00	5.00
Post-stroke Cog	18.72	5.90	0.00	32.00
Post-stroke Dep	1.99	2.03	0.00	8.00
Post-stroke ADL	0.92	1.39	0.00	5.00

*Cog, Cognition; Dep, Depression; ADL, Activity of Daily Life*.

### Longitudinal Trajectories of Depression

**Figure 1:**
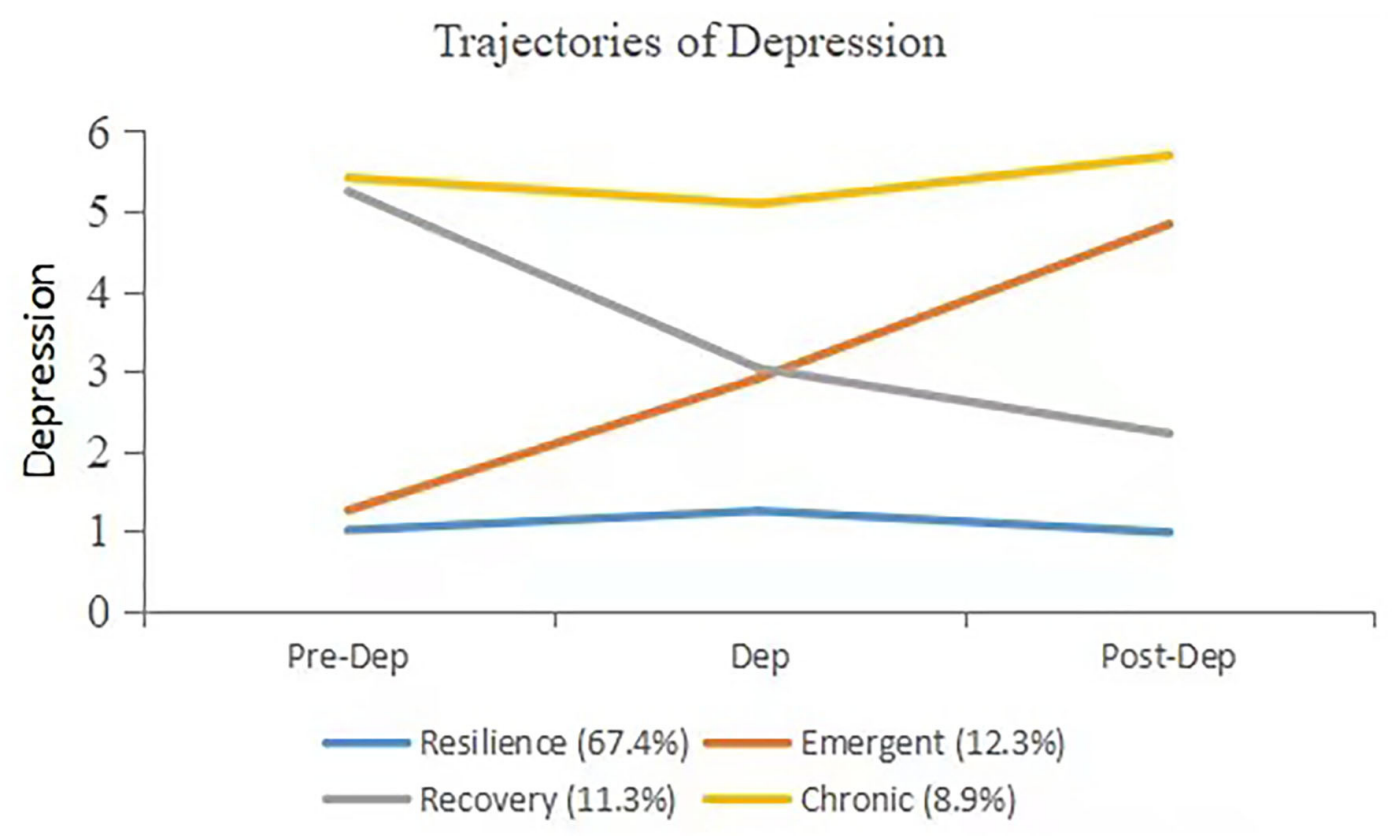
Best fitting latent growth mixture model for depression in stroke patients. Pre-dep, pre-stroke depression; Dep, depression; Post-dep, post-stroke depression.

The information criteria for the models with 2–4 classes decreased consistently, suggesting that the fit increased as the number of classes went up. The LMR LRT was significant for the two-, three-, four-class solution, but ceased to be significant for the five-class solution. Therefore, we chose the four-class solution as the best-fitting model. The entropy for the model was high (.87). As shown in Figure 1, we named the four classes as chronicity, emergent depression, recovery, and resilience. The majority of the sample (67%) was assigned to a trajectory indicative of Resilience, which was characterized by low depressive symptoms across three time points. Chronicity presented a pattern of high depression score over the time, which meant that the stroke patients suffered from depression all the way from the pre-stroke to post-stroke period. Emergent depression indicated the stroke patients reported increasing depressions scores and thus they experienced progressively severe depression over time. Recovery suggested the stroke patients' depression ameliorated over time, as the depression score decreased. Resilience referred to the group of patients who reported low depression score across three time points, which suggested that they adapted well in the face of the tragedy of stroke.

### Predicting Post-stroke Cognition

We predicted the post-stroke cognition using a hierarchical linear regression (see Table 2). In Step 1, we included gender, age, race, and ethnicity variables to control demographic differences. The model was significant, *F*(2,761) = 26.68, *p* < 0.001, *R*^2^ = 0.12. Age (*p* < 0.001, *B* = −0.24) and race (*p* < 0.001, B = −4.13) were two important predictors. In Step 2, we entered pre-cognition, which significantly improved the model, *R*^2^ = 0.44. The pre-cognition contributed noticeably to the prediction of post-cognition, *p* <.001, B = 0.70 since the base level of cognitive ability fundamentally influences its aftermath level. In Step 3, we added the three longitudinal trajectories of depression into analysis (the resilience class of stroke patients cannot be used to study the impact of depression due to its nature), *R*^2^ = 0.46. The trajectories of depression were dummy coded such that 1,1,0 represents emergent depression; 2,1,0 represents chronicity; 3,1,0 represents recovery; 4,1,0 represents resilience. The chronic (*p* < 0.001, *B* = −2.60) and emergent (*p* < 0.001, *B* = −1.63) trajectories of depression were significant. In Step 4, we further investigated the impact of pre-activity of daily living, *R*^2^ = 0.47. The pre-stroke activity of daily living is correlated with and a significant predictor of the post-cognition performance (*p* < 0.03, *B* = -0.35).

**Table 2:** Hierarchical linear regression predicting post-stroke cognition.

	**B**	**SE**	***p***	***R*^**2**^**
**Step 1**				**0.12**
Gender	−0.61	0.41	0.14	
Age	−0.24	0.03	<.001	
Race	−4.13	0.56	<.001	
Ethnicity	−1.14	1.06	0.28	
**Step 2**				**0.44**
Pre-Cog	0.70	0.03	<.001	
**Step 3**				**0.46**
Chronic	−2.60	0.59	<.001	
Emergent	−1.63	0.49	<.001	
Recovery	−0.57	0.52	0.27	
**Step 4**				**0.47**
Pre-ADL	−0.35	0.16	0.03	

*Pre-Cog, Pre-Stroke Cognition; Pre-ADL, Pre-Stroke Activity of Daily Life*.

## Discussion

In this study, we found that stroke negatively correlated depression and cognition among the geriatric population, which reaffirmed the findings of previous studies. We also found there was heterogeneity in trajectories of depression. Using the latent growth mixture modeling, we discovered four trajectories: resilience, recovery, emergent depression, and chronicity. Although the majority of individuals were resilient, there were still people who experienced chronic depression. The class of emergent depression further indicated the negative impact of stroke on patients' mental health. Controlling for demographic and pre-stroke cognition, chronicity and emergent depression were significant predictors for post-stroke cognition.

From the perspective of clinical science, our study showed that the longitudinal trajectory of depression was predictive of post-stroke cognition. This is consistent with previous findings that cognition and depression are related in the context of stroke. Our study extended these findings by including prospective data, which allowed us to evaluate the dynamics of cognition and depression pre- and post-stroke. In contrast with previous studies, we included the contribution of pre-stroke cognition into the evaluation as a baseline consideration. Subsequently, an improved examination of the reciprocally negative relationship between cognition and depression in the context of stroke resulted. Results further showed that heterogeneous depression responses to stroke were associated with post-stroke cognition. Such findings demonstrated the importance of monitoring and treating depression in cognitive remediation. The different trajectories of depression suggested that we should provide a more well-directed and appropriate treatment program for strokes patients. Instead of ignoring the depression that stroke patients suffered or just treating all classes of depression the same, classification of the specific trajectory of depression could provide the best-fit treatment plan. From the perspective of methodology, pre-stroke data served as a measure for a more accurate evaluation of the post-stroke cognition. Although the impact of stroke on depression was investigated by previous research studies, our study found that this impact was heterogeneous with latent growth mixture modeling. Different response patterns were likely to affect outcomes. Accordingly, future studies could include such means to better understand and assess the dynamics of depression and cognition.

The significance of our findings should be considered in the context of a few limitations. First, while race was a predictor for post-stroke cognition as shown in Table 2, we did not include variables that can explain the racial health disparity. Future studies should consider potential contributors to health disparity, such as health care, discrimination, and economic status. Second, the extrapolation of the relationship between cognition and depression was restricted as memory and mental status were the two mainly measured cognitive domains. Other domains, such as language, attention, and spatial orientation, were underrated in the evaluation of cognitive function and thus an aspect of improvement that future study can work on. Third, the relationship between cognition and depression among the patients who suffered from severe strokes and mortality was excluded in this study, because the measurements of cognitive ability and depression were based on survey responses that the aforementioned groups of stroke patients were not able to conduct.

In conclusion, cognition and depression experienced a mutually negative relationship; chronic and emergent depression were two statistically significant predictors of post-stroke cognition. We identified four trajectories of depression and pointed out the possibility of more specific remediation on stroke patients' depression. Future studies are encouraged to include pre-stroke cognition for better evaluation of post-stroke cognition as well as use the latent growth mixture model to testify the heterogeneous nature of depression or other variables.

## Data Availability Statement

Publicly available datasets were analyzed in this study. This data can be found here: https://hrs.isr.umich.edu/news/rand-hrs-2016-data-now-available.

## Author Contributions

The author confirms being the sole contributor of this work and has approved it for publication.

## Conflict of Interest

The author declares that the research was conducted in the absence of any commercial or financial relationships that could be construed as a potential conflict of interest.

## Publisher's Note

All claims expressed in this article are solely those of the authors and do not necessarily represent those of their affiliated organizations, or those of the publisher, the editors and the reviewers. Any product that may be evaluated in this article, or claim that may be made by its manufacturer, is not guaranteed or endorsed by the publisher.
